# Use of whole-genome sequence data for fine mapping and genomic prediction of sea louse resistance in Atlantic salmon

**DOI:** 10.3389/fgene.2024.1381333

**Published:** 2024-04-19

**Authors:** Olumide Onabanjo, Theo Meuwissen, Muhammad Luqman Aslam, Armin Otto Schmitt, Binyam Dagnachew

**Affiliations:** ^1^ Department of Animal and Aquacultural Sciences, Norwegian University of Life Sciences, Akershus, Norway; ^2^ Department of Animal Sciences, Georg August University, Göttingen, Germany; ^3^ Department of Breeding and Genetics, Nofima, Ås, Norway; ^4^ Center for Integrated Breeding Research, Göttingen, Germany

**Keywords:** Atlantic salmon, sea lice infestation, whole-genome sequence, imputation accuracy, genome-wide association study, genomic prediction

## Abstract

Sea louse (*Lepeophtheirus salmonis*) infestation of Atlantic salmon (*Salmo salar*) is a significant challenge in aquaculture. Over the years, this parasite has developed immunity to medicinal control compounds, and non-medicinal control methods have been proven to be stressful, hence the need to study the genomic architecture of salmon resistance to sea lice. Thus, this research used whole-genome sequence (WGS) data to study the genetic basis of the trait since most research using fewer SNPs did not identify significant quantitative trait loci. Mowi Genetics AS provided the genotype (50 k SNPs) and phenotype data for this research after conducting a sea lice challenge test on 3,185 salmon smolts belonging to 191 full-sib families. The 50 k SNP genotype was imputed to WGS using the information from 197 closely related individuals with sequence data. The WGS and 50 k SNPs of the challenged population were then used to estimate genetic parameters, perform a genome-wide association study (GWAS), predict genomic breeding values, and estimate its accuracy for host resistance to sea lice. The heritability of host resistance to sea lice was estimated to be 0.21 and 0.22, while the accuracy of genomic prediction was estimated to be 0.65 and 0.64 for array and WGS data, respectively. In addition, the association test using both array and WGS data did not identify any marker associated with sea lice resistance at the genome-wide level. We conclude that sea lice resistance is a polygenic trait that is moderately heritable. The genomic predictions using medium-density SNP genotyping array were equally good or better than those based on WGS data.

## 1 Introduction

In the production of Atlantic salmon, the challenge of ectoparasite infestation by sea louse (*Lepeophtheirus salmonis*) persists, causing substantial economic loss annually. This parasite feeds on the blood and tissue of salmon (Barrett et al., 2020), thus posing a significant challenge to the production, welfare, and profitability of salmon farming (Gharbi et al., 2015). Once infested, the host is predisposed to stress, anemia, stunted growth, and many other viral and bacterial infections, which may eventually lead to death (Correa et al., 2017). Benchmark Animal Health and the Norwegian Institute of Food, Fisheries, and Aquaculture Research (NOFIMA) estimated the associated losses to sea lice by the Norwegian aquaculture industry to be 6.6 billion Norwegian kroner annually (approximately 565 million euros) (Benchmark Animal Health, 2023).

To curtail this problem, some medicinal and non-medicinal methods were adopted. The extensive dependence on a few medicinal compounds due to various environmental laws resulted in sea lice building resistance against these compounds (Aaen et al., 2015). The first report of sea louse resistance to compounds such as emamectin benzoate, hydrogen peroxide, benzoyl urea, and pyrethroids in Norway was published in 2008 (Helgesen et al., 2014; Aaen et al., 2015). However, since 2017, there has been an increase in the use of non-medicinal methods such as delousing lasers, warm water dips, mechanical removal, removal using a soft brush, and plankton-shielding skirts to control sea lice. Although safer for the environment, most of these methods are stressful for salmon, affect their welfare, and, in some cases, increase post-treatment mortality rates (Overton et al., 2019). However, it has been observed that variation exists in the susceptibility of salmon to sea lice, which indicates the presence of additive genetic variance. This can be exploited by selective breeding for the genetic improvement of this trait in the population (Tsai et al., 2016). To achieve this, the genomic architecture that confers sea louse resistance to salmon needs to be dissected using genome-wide association study (GWAS). Other researchers (Tsai et al., 2016; Correa et al., 2017) have used a different number of markers (6 k–50 k SNPs) to study the association and estimate genomic breeding values for sea louse resistance. This current research differs because it uses whole-genome sequence (WGS) data to study the trait of interest. Imputation was used to infer the genotypes of missing data points and upscale ∼50 k SNPs to WGS, thereby saving costs associated with re-sequencing the genomes of thousands of samples.

The objectives of this research are to (i) estimate the imputation accuracy of genotypes in the sequenced population (with and without the inclusion of pedigree information), (ii) carry out genotype imputation for the challenged population from the array (∼50 k SNPs) to WGS, (iii) estimate the heritability of host resistance to sea lice, (iv) carry out GWAS analysis to detect quantitative trait loci (QTLs) associated with host resistance to sea lice, and (v) carry out genomic prediction and estimate the accuracy of the predicted genomic breeding values for sea louse resistance in salmon using array and WGS data.

## 2 Methods

### 2.1 Description of the populations

**FIGURE 1:**
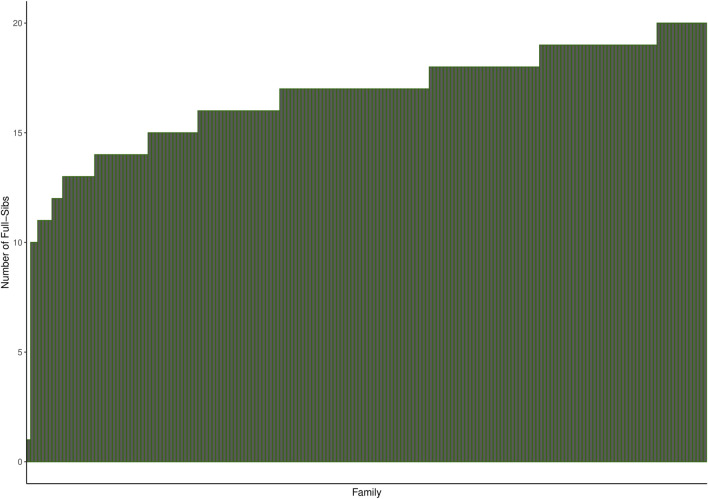
Histogram showing the number of full-sibs per family in the challenged population. The families were ordered by increasing number of full-sibs.

Mowi Genetics AS provided the phenotype and genotype (50 k SNPs) datasets for the 2017 year-class salmon population consisting of 3,185 fish of 191 full-sib families challenge-tested for sea louse infestation. The 191 full-sib families were produced by 83 sires and 182 dam, with an average of 17 full-sibs per family, as shown in Figure 1. On the other hand, the whole-genome sequencing genotype data on 197 individuals used for imputation were made available by the CMSEdit project. The sequenced data comprise individuals from the year-class 2012, 2013, 2014, 2016, 2017, and 2019 populations. The majority of the sequenced individuals were siblings of the challenged individuals belonging to the 2017 year-class, while others were related as parents and close relatives of the challenged population according to the pedigree information covering challenged and sequenced individuals provided by Mowi. Supplementary Figure S1 shows the distribution of genomic relationships for the sequenced populations, challenged population, and between both populations.

### 2.2 Challenge test data

During the challenge test, which was conducted by Mowi AS in 4 tanks at Matre, Norway, a total of 3,185 salmon smolts belonging to 191 full-sib families were infested with 45 copepodids per fish. The tank parameters were supervised and recorded, including water temperature, oxygen, and salinity. Regular monitoring was carried out daily until most of the lice reached the chalimus I stage, and the duration to reach the chalimus III stage varied from 12 to 40 days from the start of the challenge test. The fish were anesthetized upon the completion of the challenge test, and the records of sea louse count and body weight were made available for this research.

**FIGURE 2:**
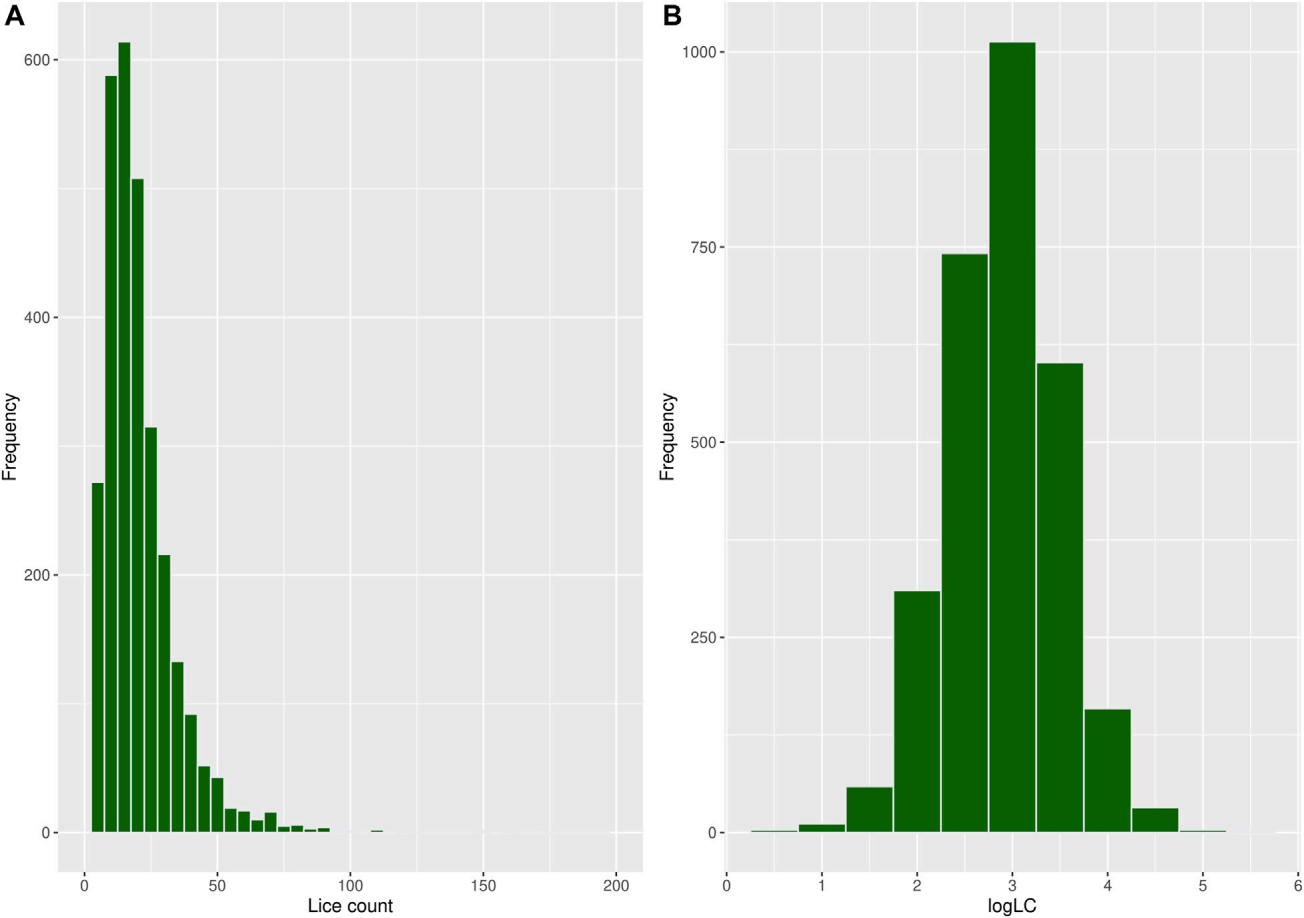
Histogram showing the frequency distribution of **(A)** louse count and **(B)** logLC of louse count.

A total of 250 observations with missing records for louse count and body weight were discarded during data cleaning, reducing the sample size to 2,935 fish with an average body weight of 109 g and a louse count of 20.5, as shown in Table 1. Most fish in the population had relatively low louse counts, while few had high counts, resulting in a right-skewed distribution, as shown in Figure 2A. A log transformation [ $\text{logLC}=\log_{e}\left(\text{sea lice count}+1\right)$ ] of these data was carried out to normalize the distribution, as shown in Figure 2B. The transformation formula adds a constant value of 1 to all sea louse counts, allowing the transformation of zero (0) sea louse count if it exists.

**TABLE 1:** Descriptive statistics of the challenged population with louse count and body weight.

	n	Mean	Median	Min	Max	Std. deviation
Louse count	2,935	20.5	17	1	262	14.44
LogLC	2,935	2.89	2.89	0.69	5.57	0.59
Body weight (g)	2,935	109.61	104.6	35	265	32.48

### 2.3 Whole-genome sequence data

The generation of whole-genome sequence data on 197 individuals and their bioinformatic sequence analysis were performed under the CMSEdit project (https://prosjektbanken.forskningsradet.no/en/project/FORISS/294504). Whole-genome resequencing was performed using the BGISEQ platform with 150 base pair paired end reads, and the raw sequence reads were trimmed and filtered using Trimmomatic (Bolger et al., 2014). Subsequently, quality sequence data were aligned to the most recent Atlantic salmon reference genome sequence (assembly Ssal_v3.1) using BWA-MEM version 0.7.13-r1126 (Li, 2013). Then, the GATK (O'Connor and van der Auwera, 2017) pipeline was used for variant discovery and genotype calling. SNPs with a minor allele frequency (MAF) lower than 5% and Mendelian errors were excluded.

The variant call data (*.vcf.gz files) were made available for this research. Quality control was performed on the detected variants, and only biallelic SNPs that had a minimum base quality of 30, genotype quality of ≥20, maximum missing rate of 30%, minimum read depth of 5, and Hardy–Weinberg equilibrium exact test (*p*-value < 10e^−25^) were included.

### 2.4 SNP genotyping

The challenge-tested fish were genotyped using the custom-developed 50 k SNP genotyping array (NOFSAL03, Affymetrix Axiom array). The SNPs across the sequence and the custom-developed 50 k SNP chip were searched for common SNPs. SNPs with a minor allele frequency lower than 5% and Mendelian errors were excluded. The chromosome level information about the number of SNPs obtained from the sequenced individuals and the overlapping SNPs to the 50 k SNP chip is summarized in Supplementary Table S1. The common SNPs found are regarded as array SNPs in the remaining parts of this paper.

### 2.5 Genotype imputation

FImpute3 software (Sargolzaei et al., 2014) was used to perform all genotype imputations as it allows for the optional inclusion of pedigree information in its imputation process. The software application uses an overlapping sliding window approach to efficiently exploit relationships or haplotype similarities between the target and reference individuals. First, the variant call format (VCF) files containing the whole-genome sequence data on the sequenced population were unzipped and then converted to the FImpute3 input format. Then, population-based genotype imputation was performed to impute data points with low-quality scores (missing genotypes) across all chromosomes.

### 2.6 Estimation of imputation accuracy

Pearson’s correlation coefficient $\left(r\right)$ was used to estimate the imputation accuracy for the sequenced population, with and without the inclusion of pedigree information. Using the k-fold cross-validation (CV) method (Refaeilzadeh et al., 2009), the sequenced population (197 individuals) was divided into 10 groups. For each CV round, all the genotypes of individuals in a validation set, except the 49,781 array SNPs, were masked, and the missing SNPs were imputed using the genotype information about the individuals in a training set. The imputed genotypes for all ten-fold were then extracted, merged, and compared to the true genotypes. The advantage of this validation method over the random sampling method is that it allows all individuals to be used to train and validate in different iterations. SNPs with poor imputation accuracy ( r < 0.6) were excluded, and the animal-based, SNP-based, and average imputation accuracy ( $\bar{r}$ ) per chromosome was estimated. The genome-wide average imputation accuracy $\left(W\right.$ ) was calculated by summing the average imputation accuracies multiplied by the number of SNPs on each chromosome and dividing by the total number of SNPs for all chromosomes. The formula is written as

$$W=\frac{\sum_{i=1}^{n}w_{i}{\bar{r}}_{i}}{\sum_{i=1}^{n}w_{i}},$$

where n is the number of chromosomes, w_i_ is the number of SNPs on chromosome i, and $\bar{r}$ _i_ is the average imputation accuracy of SNPs on chromosome i. This is necessary to avoid bias in the contributions of chromosomes to the genome-wide average imputation accuracy $\left(W\right.$ ).

### 2.7 Array to WGS imputation

After estimating the imputation accuracy, the genotype information about the sequenced individuals was used to impute the genotypes of the challenged individuals to WGS. Henceforth, WGS refers to the imputed genotypes of the individuals in the challenged population. The data were extracted and converted to PLINK raw format (Purcell et al., 2007) for further analysis.

### 2.8 Estimation of genetic parameters

The additive genetic variance, residual variance, and heritability were estimated using GCTA software (Yang et al., 2011). The genomic estimate was computed using the “--reml” command option of GCTA by implementing a univariate animal mixed model:

y=μ+Xb+Zu+e,

where **y** is the vector of the observed phenotype ( log⁡LC ), μ is the overall mean of log⁡LC , X and **Z** are assigned design matrices to the respective vectors b and u , b is the vector of fixed effects (interaction between tank*counter and body weight), **u** is the vector of random additive genetic effects with $u\;∼\;N\left(0,G\sigma_{u}^{2}\right)$ , where $\sigma_{u}^{2}$ is the additive genetic variance, and **e** is the vector of random residual effects with $e\;∼\;N\left(0,I\sigma_{e}^{2}\right)$ , where $\sigma_{e}^{2}$ is the residual variance and **I** is an identity matrix. The genomic relationship matrix (**G**) was computed according to VanRaden (2008) as

$$G=\frac{{\text{ZZ}}^{\prime }}{2*\;\sum_{i=1}^{\text{Nsnp}}p_{i}\left(1-p_{i}\right)},$$

where $p_{i}$ is the allele frequency of the second allele and $N_{\text{snp}}$ is the total number of SNP markers. The fixed effects (interaction between tank*counter and body weight) used in the model were tested against the phenotype and confirmed to be significant. The narrow sense heritability ( $h^{2}$ ) of sea louse resistance was estimated using the following formula:

$$h^{2}=\frac{\sigma_{a}^{2}}{\sigma_{p}^{2}},$$

where $h^{2}$ is the narrow sense heritability, $\sigma_{a}^{2}$ is the additive genetic variance, and $\sigma_{p}^{2}$ is the phenotypic variance.

### 2.9 GWAS

Genome-wide association analysis was conducted using GCTA software (Yang et al., 2011). This software application allows the detection of SNPs that explain a substantial proportion of the phenotypic variabilities for a complex trait. The “--mlma” command option of GCTA initiated a linear animal mixed model:

y=μ+Xb+Zu+Mα+e,

where **y** is the vector of the observed phenotype ( log⁡LC ), μ is the overall mean of log⁡LC , X and **Z** are incidence matrices to the respective vectors b and u , b is the vector of fixed effects (interaction between tank*counter and body weight), **u** is the vector of polygenic effects with $u\;∼\;N\left(0,G\sigma_{u}^{2}\right)$ , M is the incidence matrix of the candidate SNP containing marker genotypes coded as 0, 1, or 2, α is the allelic substitution effect of the candidate SNP, and **e** is the vector of random residual effects with $e\;∼\;N\left(0,I\sigma_{e}^{2}\right)$ .

The Manhattan plot was used to visualize the results, plotting ${-\;\log}_{10}\left(\text{pvalue}\right)$ against the chromosomal position of each SNP. To correct for multiple testing errors and avoid declaring non-significant SNPs as significant (false positives), Bonferroni (genome-wide and chromosome-wide threshold) correction was computed with the formulas given below:

$$\text{Genome}-\text{wide Bonferroni threshold}={\;-\;\log}_{10}\left(\frac{0.05}{N_{\text{snps}}}\right),$$

$$\text{Chromosome}-\text{wide Bonferroni threshold}={-\;\log}_{10}\left(\frac{{0.05*N}_{\text{chromosomes}}}{N_{\text{snps}}}\right).$$

SNPs whose ${-\;\log}_{10}\left(\text{pvalue}\right)$ estimate surpassed the computed thresholds were declared significant. Furthermore, a diagnostic quantile–quantile (q-q) plot was used to compare the relationship between the observed *p*-value and the expected *p*-value under the null hypothesis of no association. Both plots used functions from the qqman package (Turner, 2018) in R. The genomic inflation factor ( λ ) of the q-q plot, which provides an insight into the spurious association, was estimated using the following formula:

$$\lambda =\frac{\text{median}\left(x^{2}\right)}{0.456},$$

where x^2^ is the chi-squared test.

### 2.10 Genomic prediction

For genomic prediction, families with less than 10 siblings were excluded; this reduced the sample size from 2,935 to 2,875 individuals and full-sib families from 191 to 186. These individuals were then assigned to five folds, each comprising 575 individuals. A 5-fold within-family cross-validation genomic prediction analysis was conducted using the Bayesian generalized linear regression (BGLR) package in R (Pérez and los Campos, 2014). The “--make-rel” option in PLINK was used to fit the genomic relationship matrix (G-matrix) as covariances between animals, and the reproducing kernel Hilbert space (RKHS) model option was used in BGLR to estimate genomic breeding values. At each iteration, the adjusted phenotypes of a fold were masked and assumed unknown (validation set), while those of the other folds were not masked (training set). In this way, each observation served as training and validation at different times. Using the RKHS model, the genomic breeding value for each masked individual in the validation fold was predicted. The model used is the same as described in the “Estimation of genetic parameters” section.

### 2.11 Accuracy of genomic prediction

The accuracy of genomic prediction $\left(m\right)$ for each of the five folds was estimated by dividing Pearson’s correlation coefficient between the estimated breeding values and adjusted phenotypes by the square root of heritability. This accuracy was estimated for each of the five folds for the array and WGS data. In addition, the accuracy of all folds was estimated by extracting and merging the estimated breeding values of validation individuals for each validation fold, which makes up predicted breeding values for all samples. The predicted breeding values for all individuals were then correlated with their true adjusted phenotypes and divided by the square root of heritability. The accuracy and standard error (SE) of the folds were estimated and reported as the accuracy of the genomic prediction analysis. The formulas are represented as

$$m=\frac{\text{cor}\left(\text{EBV},y_{\text{adj}}\right)}{\sqrt{h^{2}}},$$

$${\text{SE}}_{m}=\sqrt{\frac{1-m^{2}}{n-2}},$$

where EBV is the estimated breeding values, $y_{\text{adj}}$ is the phenotype adjusted for fixed effects, $h^{2}$ is heritability, ${\text{SE}}_{m}$ is the standard error of the accuracy, $m^{2}$ is the accuracy square, and n is the number of observations.

## 3 Results

### 3.1 Imputation accuracy of WGS data

**FIGURE 3:**
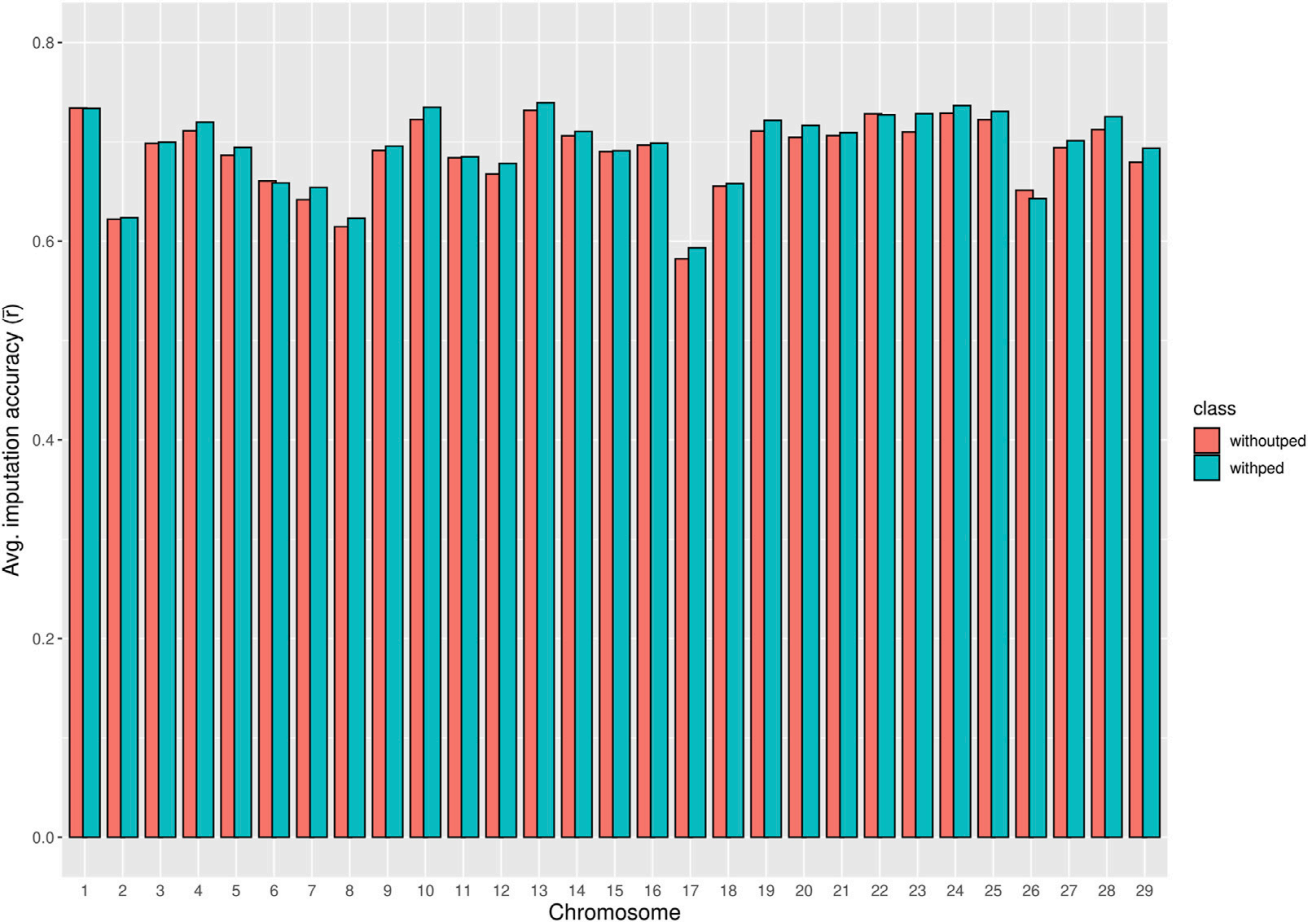
Bar plot comparing the chromosome averages of SNP-based imputation accuracy $\left(\bar{r}\right)$ with and without pedigree.

The chromosome-wise averages of imputation accuracies (animal- and SNP-based) with and without pedigree information are shown in Supplementary Table S2. As shown in Figure 3, pedigree information in the imputation process did not significantly improve the chromosome-wise SNP-based average imputation accuracy $\left(\bar{r}\right)$ . In this case, the most considerable difference between the average imputation accuracy with and without the pedigree was observed on chromosome 23, where the average accuracy with the pedigree was higher by approximately 0.02. On the other hand, the average imputation accuracy without the pedigree for chromosome 26 was higher than the average with the pedigree. Although, for most chromosomes, the average imputation accuracy with pedigree was higher than that without the pedigree, the differences were negligible.

Supplementary Table S3 shows the number of SNPs per chromosome that met the individual SNP-based imputation accuracy threshold $\left(r\geq 0.6\right)$ with and without the inclusion of the pedigree. Although the number of SNPs that met the threshold with the pedigree (3,176,724 SNPs) exceeded those without pedigree (3,141,598 SNPs), the weighted genome-wide average imputation accuracy (W) without the pedigree was highest (0.85). Therefore, it was adopted for imputation to WGS.

### 3.2 MAF and imputation accuracy

The minor allele frequencies were divided into 25 bins from 0 to 0.5 at 0.02 intervals. The average imputation accuracies of SNPs at each MAF bin were calculated and plotted against their corresponding MAF, as shown in Supplementary Figure S2. The average imputation accuracies $\left(\bar{r}\right)$ tend to increase with the minor allele frequency. The lowest average accuracy was observed on chromosome 26 with a value of 0.71 at a MAF of 0.02, while the highest average accuracy was on chromosome 25 with a value of 0.91 at a MAF of 0.46. Other observed results fell between this minimum and maximum threshold.

### 3.3 Estimation of genetic parameters

Using logLC as the phenotype, the heritability ( $h^{2}$ ) for sea louse resistance in the challenged population was estimated to be 0.21 when 49,626 array SNPs were used and 0.22 when 3,141,598 WGS SNPs were used. The additive ( $\sigma_{a}^{2}$ ) and residual variance $\left(\sigma_{e}^{2}\right)$ estimates and standard errors are also shown in Table 2.

**TABLE 2:** Estimates of genetic parameters and their standard errors for logLC .

Component	Whole-genome sequencing (WGS)	Array SNP
$\sigma_{a}^{2}$	0.061 ± 0.009	0.058 ± 0.008
$\sigma_{e}^{2}$	0.218 ± 0.007	0.223 ± 0.007
$h^{2}$	0.218 ± 0.028	0.206 ± 0.026

### 3.4 GWAS

**FIGURE 4:**
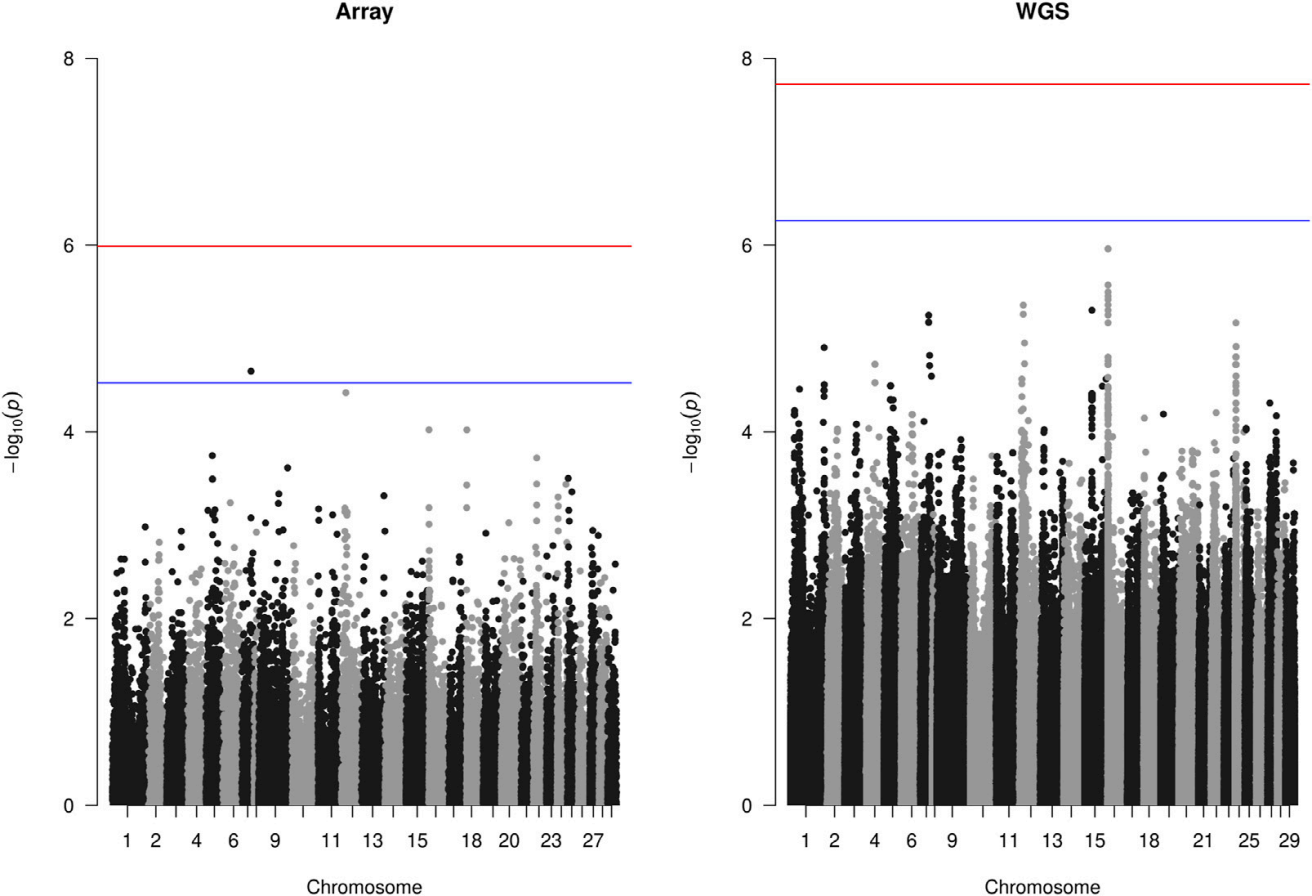
Manhattan plot of the array- and whole-genome sequence (WGS)-based genome-wide association study (GWAS) showing the ${-\log}_{10}\left(\text{pvalues}\right)$ of each SNP distributed across all autosomal chromosomes. The blue line is the chromosome-wide Bonferroni threshold, while the red line is the genome-wide Bonferroni threshold.

The GWAS analysis results for the array (49,626 SNPs) and WGS data (3,141,598 SNPs) showed no significant SNP affecting sea louse resistance. As shown in Figure 4, one SNP on chromosome 7 surpassed the chromosome-wide Bonferroni threshold (4.52) for the array data, but none reached the genome-wide Bonferroni threshold (5.99). On the other hand, none of the SNPs of the WGS data surpassed the Bonferroni chromosome-wide threshold of 6.26 or the genome-wide threshold of 7.72. For the array data, QTL signals were observed on chromosomes 5, 7, 12, 16, 18, 22, and 25, while WGS data had QTL signals on chromosomes 1, 7, 12, 15, 16, and 24. The *p*-values of the top 10 SNPs common to array and WGS data are shown in Table 3. The top 10 SNPs with the lowest *p*-values from WGS-based GWASs were unavailable on the array. Still, their high significance affirms the power of imputation in increasing the resolution in genomic regions. It is interesting to note that 8 of the 10 SNPs are within ∼60 Kbp, probably due to high LD among them.

**TABLE 3:** Top 10 SNPs according to the *p*-values of the array- and whole-genome sequence (WGS)-based genome-wide association study (GWAS).

Chr	SNP	A1	A2	MAF	Se	Array *p*-value	WGS *p*-value
***Ssa07***	HG993266.1_45156870	T	G	0.093	0.030	2.244e-05	1.955e-05
***Ssa12***	HG993271.1_26109621	T	G	0.285	0.025	3.812e-05	1.185e-04
***Ssa16***	HG993275.1_2299421	A	G	0.393	0.019	9.519e-05	1.150e-04
***Ssa18***	HG993277.2_4439352	T	C	0.383	0.018	9.543e-05	7.139e-05
***Ssa05***	HG993264.1_31252801	C	T	0.074	0.037	1.800e-04	1.193e-04
***Ssa22***	HG993281.1_16838003	G	T	0.479	0.018	1.906e-04	4.458e-04
***Ssa09***	HG993268.2_140287766	T	G	0.474	0.019	2.431e-04	2.949e-03
***Ssa25***	HG993284.1_5222628	C	T	0.385	0.018	3.141e-04	5.088e-04
***Ssa05***	HG993264.1_32056685	T	C	0.235	0.023	3.187e-04	2.192e-04
***Ssa05***	HG993264.1_32056935	T	C	0.095	0.034	3.225e-04	2.014e-04
Significant SNPs from WGS-based GWAS							
***Ssa16***	HG993275.1_2670854	T	G	0.418	0.018	-	1.095e-06
***Ssa16***	HG993275.1_2652565	T	C	0.419	0.018	-	1.096e-06
***Ssa16***	HG993275.1_2650283	C	T	0.424	0.018	-	2.676–06
***Ssa16***	HG993275.1_2695572	C	A	0.487	0.018	-	3.187e-06
***Ssa16***	HG993275.1_2666638	T	C	0.424	0.018	-	3.535e-06
***Ssa16***	HG993275.1_2695854	A	G	0.487	0.018	-	3.841e-06
***Ssa16***	HG993275.1_2698184	G	C	0.424	0.018	-	4.375e-06
***Ssa12***	HG993271.1_20073710	T	C	0.287	0.018	-	4.399e-06
***Ssa15***	HG993274.1_35023984	A	G	0.085	0.032	-	4.988e-06
***Ssa16***	HG993275.1_2671264	G	T	0.424	0.018	-	5.002e-06

*Ssa*, *Salmo salar* chromosome; A1 and A2, minor and major alleles; MAF, minor allele frequency; se, standard error.

Supplementary Figure S3 shows the quantile–quantile plot of the observed *p*-values against the expected *p*-values. It also confirms that there are no significant associations since the observed *p*-values for the top SNPs were below the expected *p*-values. The genomic inflation factor $\left(\lambda \right)$ values for the array and WGS were estimated to be 1.00 and 0.99, respectively, which confirms the absence of spurious associations.

### 3.5 Accuracy of genomic prediction

The accuracy of genomic prediction for each of the five-fold cross-validation and all folds of the array and WGS data is shown in Table 4. For the array and WGS, individuals in the third validation fold had the best prediction accuracy of 0.721 and 0.715, respectively. In comparison, the prediction accuracy for the fifth validation fold was the lowest, with a prediction accuracy of 0.578 and 0.569, respectively. Overall, using WGS for genomic predictions did not improve the accuracy of genomic prediction.

**TABLE 4:** Accuracy of genomic predictions and their standard error.

	Fold 1±SE_m_	Fold 2±SE_m_	Fold 3±SE_m_	Fold 4±SE_m_	Fold 5±SE_m_	All Folds±SE_m_
**Array**	0.676 ± 0.014	0.654 ± 0.014	0.722 ± 0.013	0.605 ± 0.015	0.578 ± 0.015	0.645 ± 0.014
**Whole-genome sequence (WGS)**	0.676 ± 0.014	0.663 ± 0.014	0.715 ± 0.013	0.587 ± 0.015	0.569 ± 0.015	0.641 ± 0.014

## 4 Discussion

### 4.1 Genotype imputation accuracies

This research reported a genome-wide average imputation accuracy ( W ) of 0.84 and 0.85 for the sequenced individuals, with and without the inclusion of pedigree information. The competitive performance of the imputation accuracy without pedigree inclusion might be because imputation methods can locate the most important haplotypes; therefore, including pedigree information did not improve our accuracy. This report is contrary to those of earlier studies (Huang et al., 2012; Sargolzaei et al., 2014) that reported an increase in imputation accuracy with the inclusion of pedigree information consisting of close relatives. Manousi (2021) assessed the effect of the new Atlantic salmon genome assembly on the imputation accuracy by comparing the imputation reliability $\left(r^{2}\right.$ ) of Beagle (Browning and Browning, 2009) and FImpute3 (Sargolzaei et al., 2014). Imputation reliabilities ( $r^{2}$ ) of 0.85 and 0.83 were reported for Beagle and FImpute3 (with pedigree), respectively, after imputing from 44 k to 440-k SNP density. This translates to Pearson’s correlation coefficient ( r ) values of 0.92 and 0.91, respectively, which are higher than 0.84 (with pedigree) reported here.

In addition, Yoshida et al. (2018) reported the imputation accuracy ( r ) ranging between 0.74 and 0.98, having tested different imputation scenarios. They performed genotype imputation to 50 k SNPs using FImpute2.2 software (Sargolzaei et al., 2014) with varying SNP densities (500, 3 k, and 6 k) and varying numbers of reference and validation animals in the Atlantic salmon population. Our findings agree with the imputation accuracy range found by Yoshida et al. (2018), although the sample size and number of SNPs used were lower. Kijas et al. (2017) used a multi-generation reference population of Tasmanian Atlantic salmon to carry out imputation from 5 k to 78 k. They reported a high genotype imputation accuracy of 0.89–0.97, while Tsai et al. (2017) reported an imputation accuracy ( r ) of 0.62–0.90 in UK-farmed salmon.

It should be noted that the accuracies reported for the sequenced population in this study do not infer the imputation accuracy of the challenged population that was imputed to WGS as the true sequence genotypes of these individuals would be required to estimate their accuracy. However, the average genomic relationship between the challenged and sequenced populations and within the sequenced population is very similar, as shown in Supplementary Figure S1, suggesting that the imputation accuracy derived from sequenced and challenged individuals might be close to the accuracy of imputation in large datasets. The imputation accuracies reported in all these salmon studies were relatively high, further confirming the relevance of genotype imputation in saving costs relating to high-density genotyping or re-sequencing of a large number of animals in the aquaculture industry.

### 4.2 Relationship between the MAF and imputation accuracy

The relationship between the MAF and imputation accuracy was observed by dividing SNPs into 25 bins according to their MAF. The average imputation accuracy ( $\bar{r}$ ) is the average of Pearson’s correlation $\left(r\right)$ between the true and the imputed genotypes for SNPs belonging to a particular bin. Imputation accuracy increased with an increase in MAF, which corresponds with the findings of Pausch et al. (2013), Tsai et al. (2017), and Jiang et al. (2022). They all reported increasing imputation accuracy for known variants.

Although it was observed that the imputation accuracy slightly decreased in some bins as the MAF increased, this could be due to the low number of SNPs in those bins, resulting in sampling errors.

### 4.3 Genetic parameters

The heritability of sea louse resistance in the population of Norwegian Atlantic salmon studied in this research was estimated to be 0.21 and 0.22 for array and WGS data, respectively. These findings are consistent with the reports obtained by several researchers. Aslam et al. (2023) studied salmon belonging to year-classes 2018 and 2022 from the Mowi Genetics population, and they reported a heritability of 0.25 and 0.20. In addition, Fraslin et al. (2023) studied three different year classes (2017–2019) of salmon from the Benchmark genetics population. Salmon from each year class was divided into two, raised, and challenge-tested with the predominant lice species in two locations (Chile and Iceland). They reported heritability ranging from 0.10 to 0.21 for salmon challenged with *L. salmonis* in Iceland and 0.15 to 0.26 for salmon challenged with *Caligus rogercresseyi* in Chile.

Furthermore, Gharbi et al. (2015) reported a heritability of 0.30 in a Scottish salmon population. Rochus et al. (2018) reported an estimated heritability of 0.29 when louse count phenotype data were log-transformed and 0.17 when they were not. Some low heritability values have also been reported for host resistance to sea lice. Correa et al. (2017) reported an estimated heritability of 0.12, while Kjetså et al. (2020) and Odegård et al. (2014) estimated the heritability of sea louse resistance to be 0.14.

The differences observed in the heritability estimated and reported by various researchers could be due to the species of salmonoid and sea lice studied, phenotype transformation, the difference in population or year class, the type of model used, the type of challenge tests (land-based or sea cages) carried out, experimental design, and pedigree *versus* genomic estimates. All heritability estimates reported in these various studies fall in the low-to-moderate heritability range and, therefore, suggest that the trait of interest can be improved by selective breeding.

### 4.4 GWAS

GWASs employ a statistical approach to map variants (from SNP arrays or WGS data) associated with traits of interest (The Wellcome Trust Case Control Consortium, 2007). This requires the availability of genotypes of thousands to millions of variants and phenotypes for a reasonable number of individuals within a population (Altshuler and Daly, 2007). In humans, this method has identified close to 200,000 SNPs associated with complex traits and diseases (Buniello et al., 2019).

Our findings of the association test using the array and WGS data in this study did not identify any SNP on the genome-wide level to be in association with the sea louse resistance trait, indicating that the trait is likely polygenic. Although one SNP on chromosome 7 of the array was observed to have a chromosome-wide significance, none was observed in WGS data. In addition, it was observed that both array and WGS scenarios had strong signals on chromosomes 7, 12, and 16. If these regions are studied, they might harbor putative genes that affect sea louse resistance.

Our result of no significant genome-wide QTL for sea louse resistance agrees with the findings of Tsai et al. (2016), Correa et al. (2017), and Fraslin et al. (2023). On the other hand, Aslam et al. (2023) recently found significant QTLs for sea louse resistance after studying 2 year classes (2018 and 2022) of salmon, with the latter year class being offspring of the former. They reported strong signals across chromosomes 2, 5, 11, and 25. In addition, Rochus et al. (2018) studied louse resistance in the North American salmon population. They used the forward multiple linear regression and a linear mixed model and detected QTLs on different chromosomes. The latter identified two QTLs located on chromosomes 1 and 23, respectively, while the former identified 70 SNPs, many of which might be due to not correcting for the population structure. The differences in reports could be due to the sample size, population of salmon studied, population structure, and the type of challenge test (land-based or sea cages).

The similar outcome of both GWAS scenarios as against our expectations of the better performance of WGS data might be due to various reasons. One such reason could be due to the over-correction of effects, which was put in place to avoid spurious associations. As shown in Supplementary Figure S3, our lambda values were a bit deflated. Another reason could be due to the strict Bonferroni correction method adopted in our analysis to correct for multiple testing problems. This method divides the adopted *p*-value (0.05 in this case) by the total number of SNPs, which is over 3 million for WGS. This results in a genome-wide significance threshold that might be difficult for any SNP to surpass. Therefore, considering the close relationships in these populations, the 50 k array data would perform optimally, and imputation to higher densities might be necessary in a more distant population.

### 4.5 Accuracy of genomic prediction

Over the years, genomic selection (Meuwissen et al., 2001) has proven to be a valuable tool for the improvement of animal populations. It became popular and widely adopted after the advent of affordable genome-wide SNP chips in 2008 and has provided a lasting solution to traditional breeding problems such as long generation intervals, slow genetic gain, and the high cost of maintaining animals. This methodology allows for the selection of progenies with good estimated breeding values using phenotype and genotype information about parents and other closely related animals. Therefore, it is paramount to accurately predict estimated breeding values to select the best offspring that will be the parents of the next generation for the continuous improvement of the animal population.

This study reported the accuracy of a 5-fold within-family cross-validation scheme for genomic prediction. The assessment of each fold showed that for the array and WGS data, individuals in the third fold had the best prediction accuracy of 0.722 and 0.715, respectively. In contrast, individuals in the fifth fold had the lowest prediction accuracy of 0.578 and 0.569. The variation observed in the prediction accuracy across folds may be due to the variation in the number of sibs per family. Some families had more sibs in the training fold than others; therefore, the breeding values of their sibs in the validation set were predicted more accurately. This finding agrees with the conclusion obtained by Fraslin et al. (2022), who studied the impact of the genetic relationship between the training and validation populations in Atlantic salmon. They concluded that a close genetic relationship between training and validation individuals enhances the accuracy of genomic prediction.

Tsai et al. (2017) performed a 5-fold cross-validation and reported the estimated genomic prediction accuracy of 0.58 and 0.60 for sea louse resistance when using imputed and true genotypes. In addition, Aslam et al. (2023) reported an accuracy of 0.60 and 0.58 for the 2 year classes studied, while Fraslin et al. (2022) reported a prediction accuracy of 0.39 and 0.49 for the 2010 and 2014 populations, respectively. Tsai et al. (2017) and Fraslin et al. (2022) estimated the accuracy by dividing the correlation between the predicted genomic breeding values and the phenotype by the square root of heritability. In contrast, this study estimated the accuracy by dividing the correlation between the predicted genomic breeding values and the adjusted phenotypes by the square root of heritability. This formula using the adjusted phenotype for fixed effects rather than the unadjusted phenotype is better because the unadjusted phenotype consists of fixed, random effects of animals and residuals. Therefore, estimating the accuracy by calculating the correlation between the estimated breeding values and phenotypes provides a lower accuracy estimate. Using the adjusted phenotype provides better accuracy as it gets us closer to estimating the effect of markers.

One might expect that using the WGS data would provide a higher accuracy of genomic prediction accuracy than the 50 k SNP array, but this is not the case for the result presented herein. In fact, the accuracy of genomic prediction for all validation with the array was slightly higher than that with the WGS (0.645 and 0.641, respectively). This is because aquaculture species unlike other farmed species are characterized by high fecundity that provides aquaculture breeding programs with thousands of full-sib families for sibling-testing schemes. These siblings share large genomic segments that are effectively captured by low- and medium-density (5,000) SNPs, and the estimate of genomic prediction using these SNPs has been reported to be as effective as using high-density panels (Tsai et al., 2016; Vallejo et al., 2017; Robledo et al., 2018; Yoshida et al., 2018). However, this high accuracy using a low-to-medium number of SNPs might only be observed in sibling-testing schemes. When the relationship between the training and validation populations is more distant, a higher SNP density would provide better prediction accuracy (Tsai et al., 2016; Tsairidou et al., 2020). Therefore, it could be more advantageous to impute from low-(500)-to-medium density (5,000) SNPs or medium-(5,000)-to-high density (50 k) SNPs for genomic prediction in aquaculture species.

## 5 Conclusion

The inclusion of pedigree information in genotype imputation did not improve the average genome-wide imputation accuracy. Host resistance to sea lice is a moderately heritable trait that can be improved with selective breeding. The high signals observed across the chromosomes with no significant associated QTL detected confirm the polygenic nature of host resistance to sea lice. Finally, the 50 k SNP data for this study were sufficient to conduct GWAS analysis and accurately predict genomic breeding values for sea louse resistance trait.

## Data Availability

The datasets presented in this article are not readily available because they are co-owned by a third party. Requests to access these datasets should be directed to binyam.dagnachew@nofima.no.
