# Supplementary material for: Use of whole-genome sequence data for fine mapping and genomic prediction of sea louse resistance in Atlantic salmon
Source: Front Genet. 2024 Apr 19;15:1381333. doi: 10.3389/fgene.2024.1381333 (PMC11066268; doi:10.3389/fgene.2024.1381333)
Supplement: Supplementary file 1 [file Table1.pdf]

Supplementary Table 1: Summary of SNPs common to the whole-genome sequencing and the custom-developed 50k SNP chip

Chromosome	Chromosome size (bp)	Number of whole-genome sequence SNPs	Number of common Array SNPs
Ssa01	174,498,729	315,203	3,636
Ssa02	95,481,959	154,223	1,604
Ssa03	105,780,080	196,962	2,393
Ssa04	90,536,438	168,726	1,995
Ssa05	92,788,608	156,403	2,016
Ssa06	96,060,288	175,325	1,953
Ssa07	68,862,998	123,674	1,404
Ssa08	28,860,523	44,976	418
Ssa09	161,282,225	258,770	2,744
Ssa10	125,877,811	211,792	2,585
Ssa11	111,868,677	176,679	1,922
Ssa12	101,677,876	189,122	2,000
Ssa13	114,417,674	196,755	2,502
Ssa14	101,980,477	171,528	2,215
Ssa15	110,670,232	195,975	2,045
Ssa16	96,486,271	151,977	1,693
Ssa17	87,489,397	108,117	1,156
Ssa18	84,084,598	142,757	1,376
Ssa19	88,107,222	153,362	1,600
Ssa20	96,847,506	162,802	2,007
Ssa21	59,819,933	117,562	1,185
Ssa22	63,823,863	122,674	1,449
Ssa23	52,460,201	110,988	1,386
Ssa24	49,354,470	93,267	1,205
Ssa25	54,385,492	99,906	1,171
Ssa26	55,994,222	98,126	1,012
Ssa27	45,305,548	98,869	1,202
Ssa28	41,468,476	87,008	1,015
Ssa29	43,051,128	91,370	892
**Total**	**2,499,322,922**	**4,374,898**	**49781**

Ssa stands for *Salmon salar*, and ***01 to 29*** represent the respective autosomal chromosomes.
