# Supplementary material for: Use of whole-genome sequence data for fine mapping and genomic prediction of sea louse resistance in Atlantic salmon
Source: Front Genet. 2024 Apr 19;15:1381333. doi: 10.3389/fgene.2024.1381333 (PMC11066268; doi:10.3389/fgene.2024.1381333)
Supplement: Supplementary file 2 [file Table2.pdf]

Supplementary Table 2: Average of SNPs and animal-based imputation accuracies (all SNPs)

**Chromosomes**	**Avg. of Imputed SNPs Accuracy (without pedigree)**	**Avg. of Imputed SNPs Accuracy (with pedigree)**	**Avg. of Animal-Based Accuracy (without pedigree)**	**Avg. of Animal-Based Accuracy (with pedigree)**
Ssa01	0.734	0.734	0.842	0.842
Ssa02	0.622	0.624	0.769	0.768
Ssa03	0.698	0.700	0.821	0.820
Ssa04	0.711	0.720	0.826	0.832
Ssa05	0.686	0.694	0.816	0.820
Ssa06	0.661	0.659	0.794	0.794
Ssa07	0.642	0.654	0.782	0.785
Ssa08	0.615	0.623	0.761	0.771
Ssa09	0.691	0.696	0.816	0.817
Ssa10	0.723	0.735	0.834	0.842
Ssa11	0.684	0.685	0.811	0.811
Ssa12	0.668	0.678	0.792	0.799
Ssa13	0.732	0.739	0.839	0.844
Ssa14	0.706	0.710	0.824	0.827
Ssa15	0.690	0.691	0.817	0.815
Ssa16	0.697	0.699	0.818	0.821
Ssa17	0.582	0.593	0.742	0.753
Ssa18	0.656	0.658	0.793	0.793
Ssa19	0.711	0.722	0.825	0.834
Ssa20	0.705	0.717	0.823	0.833
Ssa21	0.706	0.709	0.817	0.818
Ssa22	0.728	0.727	0.835	0.831
Ssa23	0.710	0.728	0.824	0.833
Ssa24	0.729	0.737	0.839	0.842
Ssa25	0.722	0.731	0.834	0.840
Ssa26	0.651	0.643	0.785	0.784
Ssa27	0.694	0.701	0.816	0.820
Ssa28	0.712	0.725	0.822	0.829
Ssa29	0.679	0.694	0.800	0.807
**Weighted Average**	**0.692**	**0.698**	**0.811**	**0.815**
