# Supplementary material for: Use of whole-genome sequence data for fine mapping and genomic prediction of sea louse resistance in Atlantic salmon
Source: Front Genet. 2024 Apr 19;15:1381333. doi: 10.3389/fgene.2024.1381333 (PMC11066268; doi:10.3389/fgene.2024.1381333)
Supplement: Supplementary file 3 [file Image2.pdf]

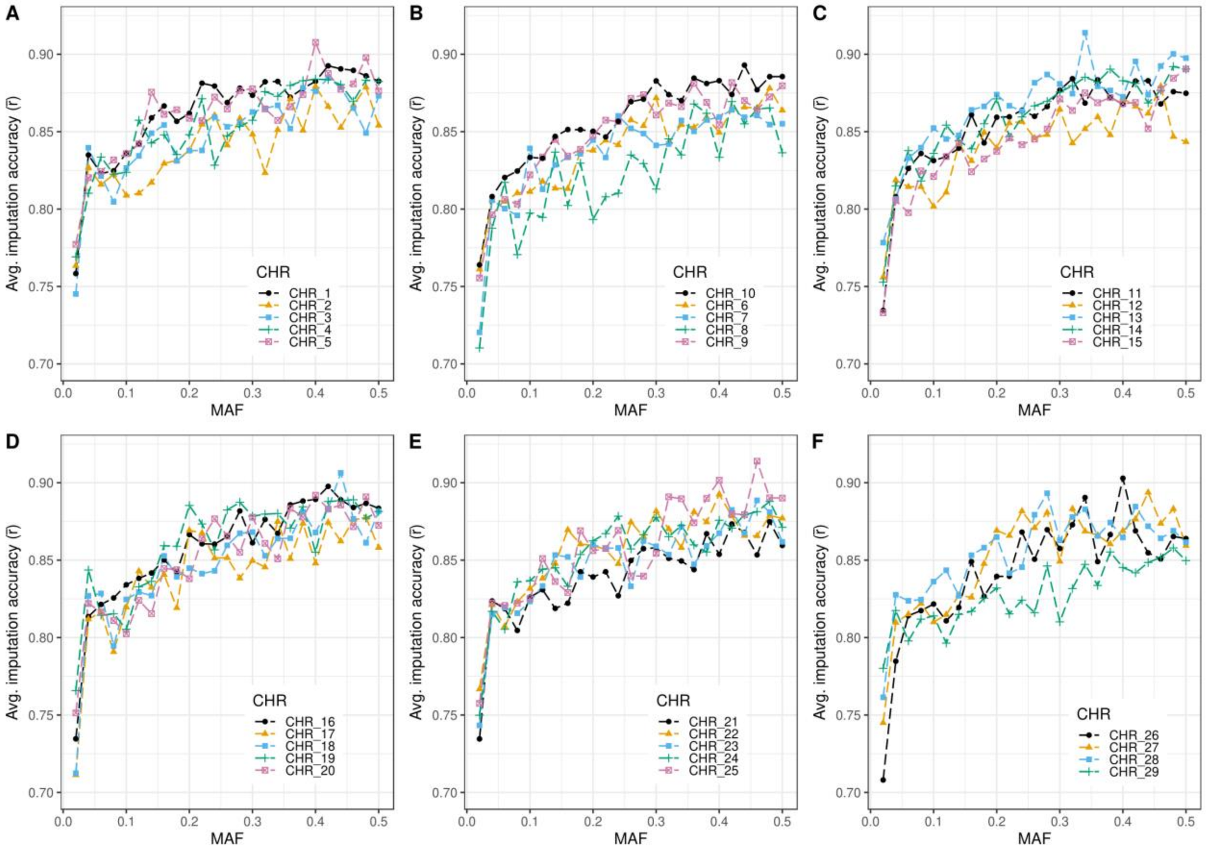

Supplementary Figure 2: Plots showing the average imputation accuracy ($\bar{r}$) against the minor allele frequency (MAF) bin of imputed SNPs for all autosomal chromosomes.
