# Supplementary material for: Use of whole-genome sequence data for fine mapping and genomic prediction of sea louse resistance in Atlantic salmon
Source: Front Genet. 2024 Apr 19;15:1381333. doi: 10.3389/fgene.2024.1381333 (PMC11066268; doi:10.3389/fgene.2024.1381333)
Supplement: Supplementary file 4 [file Image3.pdf]

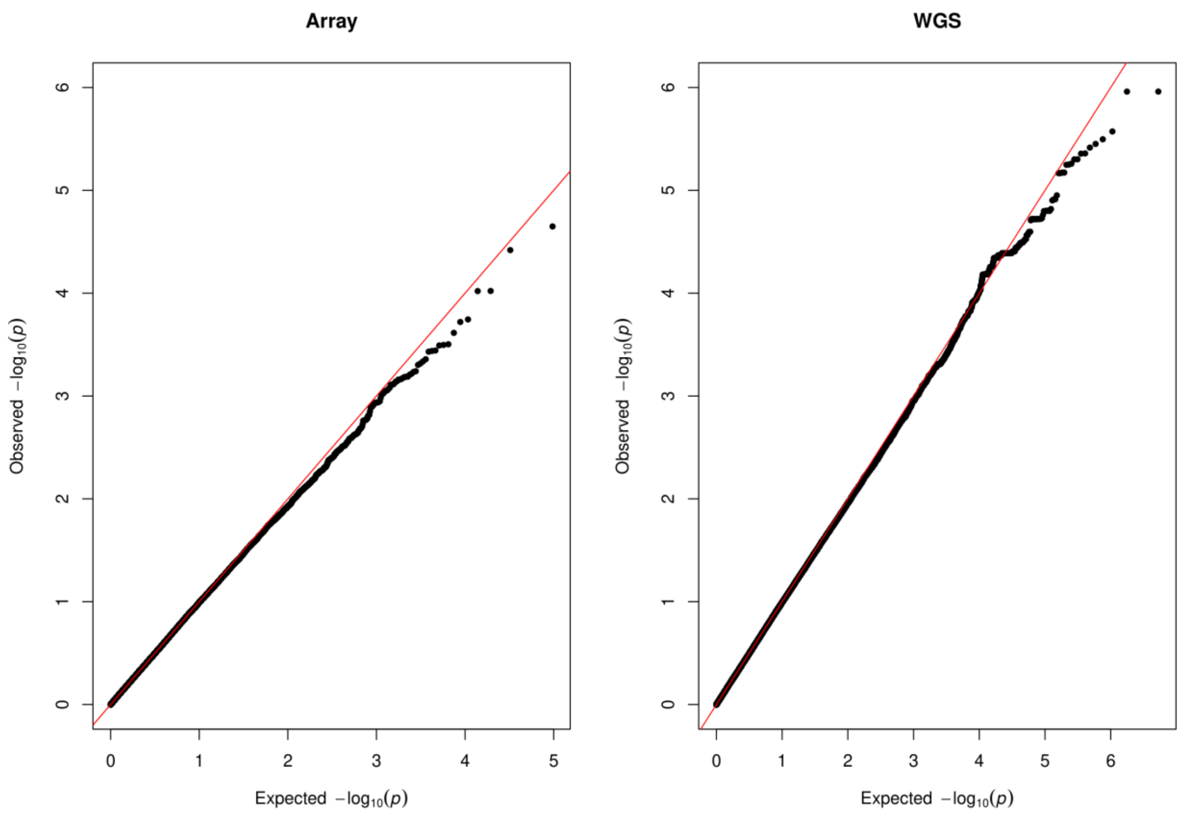

Supplementary Figure 3: Quantile-quantile plot showing observed against expected $-\log_{10}$(pvalues) for array and WGS. The red diagonal line indicates the null hypothesis of no association.
