# Supplementary material for: Use of whole-genome sequence data for fine mapping and genomic prediction of sea louse resistance in Atlantic salmon
Source: Front Genet. 2024 Apr 19;15:1381333. doi: 10.3389/fgene.2024.1381333 (PMC11066268; doi:10.3389/fgene.2024.1381333)
Supplement: Supplementary file 5 [file Table3.pdf]

Supplementary Table 3: Averages of SNP-based imputation accuracy after excluding poorly supplementary

Chromosomes	Sequence	Array SNPs	Selected SNPs (r >= 0.6) without pedigree	Selected SNPs (r >= 0.6) with pedigree	Average accuracy ($\bar{r}$) without pedigree	Average accuracy ($\bar{r}$) with pedigree
Ssa01	315,203	3626	242209	241853	0.862	0.859
Ssa02	154,223	1586	96549	96866	0.837	0.827
Ssa03	196,962	2377	144037	144788	0.846	0.841
Ssa04	168,726	1993	126719	127886	0.850	0.851
Ssa05	156,403	2007	111493	112816	0.856	0.853
Ssa06	175,325	1936	119769	119666	0.832	0.826
Ssa07	123,674	1399	82372	84412	0.835	0.831
Ssa08	44,976	410	29014	29603	0.819	0.821
Ssa09	258,770	2738	187277	189755	0.842	0.836
Ssa10	211,792	2574	161101	164418	0.851	0.852
Ssa11	176,679	1913	125288	124784	0.852	0.847
Ssa12	189,122	1995	128315	131180	0.837	0.837
Ssa13	196,755	2499	149951	151568	0.862	0.862
Ssa14	171,528	2209	124349	125445	0.854	0.850
Ssa15	195,975	2044	140291	140565	0.841	0.834
Ssa16	151,977	1680	109846	110589	0.854	0.846
Ssa17	108,117	1154	64048	65661	0.840	0.835
Ssa18	142,757	1373	96174	96598	0.846	0.842
Ssa19	153,362	1599	112502	115062	0.855	0.856
Ssa20	162,802	2007	118256	120868	0.847	0.848
Ssa21	117,562	1184	86732	87858	0.837	0.830
Ssa22	122,674	1449	93358	93664	0.853	0.846
Ssa23	110,988	1386	82481	85395	0.846	0.845
Ssa24	93,267	1205	71355	72363	0.853	0.850
Ssa25	99,906	1170	75645	76774	0.853	0.851
Ssa26	98,126	1004	64531	62794	0.841	0.835
Ssa27	98,869	1202	70201	71100	0.848	0.845
Ssa28	87,008	1015	63665	66145	0.850	0.843
Ssa29	91,370	892	64070	66248	0.823	0.823
**Total**	**4374898**	**49626**	**3141598**	**3176724**	**0.848**	**0.844**
