# Supplementary material for: Use of whole-genome sequence data for fine mapping and genomic prediction of sea louse resistance in Atlantic salmon
Source: Front Genet. 2024 Apr 19;15:1381333. doi: 10.3389/fgene.2024.1381333 (PMC11066268; doi:10.3389/fgene.2024.1381333)
Supplement: Supplementary file 6 [file Image1.pdf]

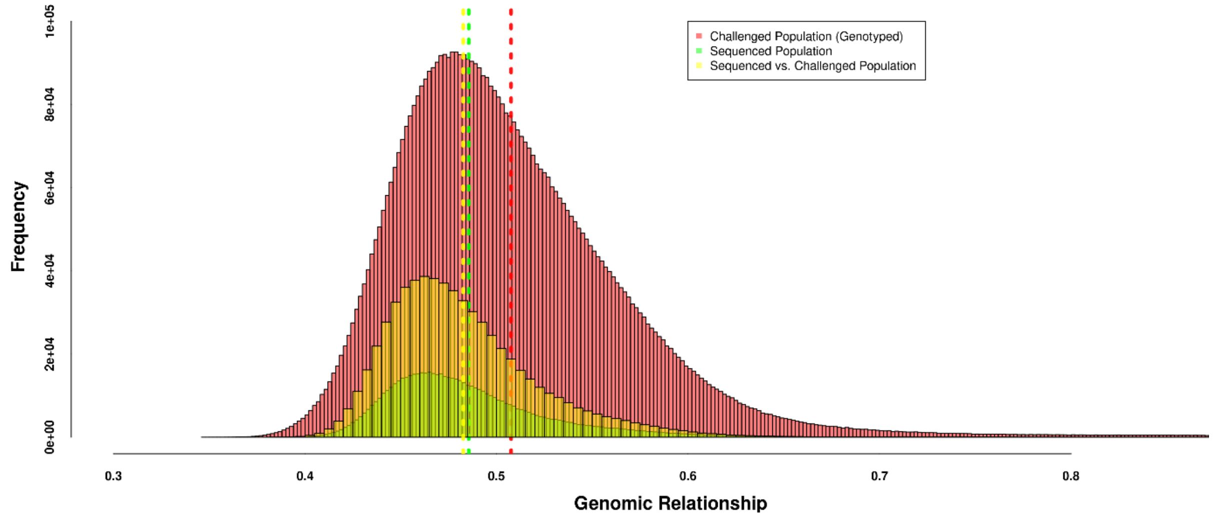

Supplementary Figure 1: Distribution of off diagonal elements of genomic relationships for sequenced populations, challenged population, and between both populations. The dashed vertical lines are the mean relationship of each group. The genomic relationship matrix was computed using GCTA with all allele frequency fixed to 0.5. This is because the data comprises of individuals from different year classes with different allele frequencies.
